# Mental Disorders Among Healthcare Workers During the Coronavirus Pandemic

**DOI:** 10.7759/cureus.54537

**Published:** 2024-02-20

**Authors:** Jorge I Zurutuza-Lorméndez, Liliana Ovando-Diego, Marco A Lezama-Prieto, Jaime Morales-Romero, Christian S Ortiz-Chacha, Santiago Gonzalez-Periañez

**Affiliations:** 1 Epidemiology and Biostatistics, Centro de Investigaciones Biomédicas, Universidad Veracruzana, Xalapa, MEX; 2 Family Medicine, Unidad de Medicina Familiar No. 66, Instituto Mexicano del Seguro Social, Xalapa, MEX; 3 Family Medicine, Unidad de Medicina Familiar No. 27, Instituto Mexicano del Seguro Social, Papantla, MEX; 4 Epidemiology and Public Health, Instituto de Salud Pública, Universidad Veracruzana, Xalapa, MEX; 5 Chemistry, Centro de Investigaciones Biomédicas, Universidad Veracruzana, Xalapa, MEX

**Keywords:** covid-19, healthcare workers, psychological exhaustion, depressive disorder, anxiety disorders

## Abstract

Introduction:Coronavirus disease 2019 (COVID-19) is a disease that has become a regular part of care by health services. In the beginning, health services faced immense pressure due to new disease exposure, irregular schedules, and high work stress for healthcare workers. Unfortunately, their mental health was not adequately safeguarded, and there are few healthcare units that screen staff for depression and anxiety.

Objective: The objective of this study is to describe the prevalence and factors associated with anxiety and depression diagnoses among healthcare workers during the coronavirus pandemic.

Materials and methods: A cross-sectional study was conducted in which depression (Beck questionnaire) and anxiety (Hamilton questionnaire) were investigated in health staff, after providing informed consent. This study was carried out during November and December 2022. All workers in all areas of a first-level unit were invited to participate in this research, so no sample calculation or sampling technique was required. Statistical analysis was performed using X^2^ and Student’s t-test.

Results: Among the 232 workers surveyed, the prevalence of mild anxiety, severe anxiety, and certain levels of depression was 42.1%, 33.5%, and 18.9%, respectively. The study revealed that smoking is associated with a higher risk of anxiety diagnosis (OR=4; CI_95%_=1.3-12.7). A higher score on the Hamilton Anxiety Scale (OR=1.07; CI_95%_=1.04-1.11) as well as not being permanent staff (OR=3.34; CI_95%_=1.2-9.3) was found associated with depression diagnosis.

Conclusion: The SARS-CoV-2 pandemic increased the stress and pressure on healthcare workers. Thus, early detection, timely treatment, and effective prevention measures are necessary for safeguarding health status and the provision of healthcare services.

## Introduction

Although anxiety and depression are common pathologies in the world population, their prevalence in an entire country is unclear due to multiple conditions that affect them, such as the economic, social, and cultural circumstances of the people, as well as individual personality and biological susceptibility.

Some studies showed that the prevalence of depression in Mexico in 2018 was close to 13.3%, and for anxiety, it was around 32.6% in 2020; the most frequent risk factors for both diseases are female gender, unemployment, and low socioeconomic level [1,2]. Nevertheless, these diseases, which have become relevant in recent years, are also affected by global events like the coronavirus disease 2019 (COVID-19) pandemic, putting additional pressure on the population, especially on healthcare workers [3]. 

It is not the first time that the world has faced a coronavirus with high mortality and pandemic potential. In 2002, there was an outbreak of Severe Acute Respiratory Syndrome caused by a coronavirus (SARS-CoV) in China, and in 2012 we faced Middle East Respiratory Syndrome (MERS-COV) in Saudi Arabia; these had a high mortality rate for respiratory complications, and these diseases were controlled with efforts of the affected countries and the international community [4]. The SARS-CoV-2 has put a strain on health systems around the world. Its rapid expansion and high capacity to cause serious diseases modify the healthcare response, particularly since early identification is extremely difficult [5].

For Mexico, in January 2023, there were 7,342,764 cases and 331,897 confirmed deaths were reported, with a mortality rate of 4.05% during this period [6]. In order to avoid high incidence and mortality, hospital conversion was attempted, which consists of increasing the number of useful beds to treat severe forms of the disease and increasing the capacity of existing units to treat the burden of other diseases [7]. However, it was not possible to increase the number of physicians, and even several who had chronic illnesses had to take shelter.

The staff of health institutions (non-clinical staff) and healthcare workers (medical, nursing, laboratory, and imaging personnel) are experiencing uncertainty in providing care in a country with a high prevalence of obesity and chronic degenerative diseases, which are important risk factors for mortality [8-10]. This, coupled with the absence of protective equipment to avoid infection, raises concerns among healthcare workers about getting infected [11].

During the pandemic, the speed at which information about COVID-19 was spread, as well as the constant changes in public policy in Mexico, caused confusion and mistrust among the Mexican population [12]. This, combined with the isolation methods hospitals used for severe cases, posed challenges for patient-centered care [13]. These challenges were particularly burdensome for the Mexican health system and caused an extreme emotional toll on healthcare workers [14-17].

When the influenza (H1N1) pandemic occurred in Mexico in 2009, healthcare workers suffered a significant increase in psychological stress related to concerns about their family’s health, doubts about their functional abilities, and fear of stigmatization. On top of that, they had to cope with work stress, social isolation, and concerns about their health. Other risk factors reported in the literature include the constant change of information and procedures, media scrutiny, being part of the nursing staff, increased perception of individual risk, lifestyle changes due to the disease, and personal vulnerability; all of this caused many medical personnel to not go to work in 2009, despite their strong sense of duty. The most frequent problems that have been reported in the literature are post-traumatic stress syndrome, anxiety, depression, and burn-out syndrome [18].

The Mexican Social Security Institute (or IMSS per its Spanish acronym for Instituto Mexicano del Seguro Social) was the public institution in Mexico that established guidelines in 2021 to protect the mental health of the workers at the units. However, the efforts were focused on treating patients and controlling the spread of COVID-19. Although these support mechanisms were available for workers, it is not known whether they could be implemented completely and correctly or their beneficial effects [19].

Evaluation and measurement of mental illnesses, such as anxiety and depression, are crucial in the workplace to prevent health issues that can affect job performance. Mental health alterations can have both physiological and cognitive effects; the latter can lead to behavioral and emotional disorders. Various studies describe prevalences of these conditions in health personnel as high as 50% for depression and 46% for severe anxiety [20]. The overwhelming demand for urgent healthcare during the COVID-19 pandemic has created a stressful environment for workers, leading to feelings of tension, helplessness, and constant frustration. If left unaddressed, these issues can have harmful physical and mental health consequences and can even be fatal, if are not prevented and attended on time.

Therefore, our study aimed to identify the prevalence of personnel with a diagnosis of mental illnesses and the risk factors associated, which can be used in the future to further analyze more effective ways to support them by implementing better strategies for diagnostics, complying with more comprehensive care from the staff themselves. Likewise, we will be able to identify the specific needs of workers and find the most effective way to care for them. In the present study, the prevalence of anxiety and depression was identified among health personnel from different medical and paramedical areas, during the pandemic caused by the COVID-19 disease at a Family Medical Unit (or UMF, per its Spanish acronym for Unidad de Medicina Familiar) of the IMSS, in Xalapa, Veracruz, Mexico.

## Materials and methods

A cross-sectional study was conducted, which included clinical and non-clinical staff from a primary care unit of the IMSS. The inclusion criteria were personnel assigned to the UMF who worked for at least six months from January to December 2022, personnel who were in direct care with patients and, therefore, being at risk for SARS-CoV-2 infection within the UMF. Personnel working for any other public institution other than the IMSS were excluded. The exclusion criteria were personnel who decided not to complete the required questionnaires or decided that their data would be excluded from the research, and personnel who lied about their sociodemographic or work characteristics during data collection. Incorrect or incomplete forms were eliminated from the final grouping to avoid invalidating our study. Of the 490 workers at the unit, 232 met the selection criteria and all were included. There was no dropout. The research was approved by the Institutional Ethics and Research Committee. Our study was carried out between November and December 2022, using the Hamilton Anxiety Rating Scale (HAS) and Beck Depression Inventory (BDI) to explore anxiety and depression, respectively [21,22]. Also, the Graffar-Méndez-Castellanos socioeconomic tool was used to complement this work [23]. We conducted a survey to gather data on socio-demographic information, comorbidities, weight, and height of the workers. Additionally, a set of 19 Likert-type questions were asked where the workers indicated their level of agreement about their attitude toward COVID-19.

Continuous variables between two independent groups were compared using the Student’s t-test and ANOVA for three or more independent groups. The *chi*-square test was used to compare categorical variables between independent groups. All the statistical tests were two-tailed. Correlation analyses through Spearman’s rank correlation coefficient were performed to identify the relationship between the tool scores. The risk factors were identified by calculating the odds ratio (OR) and their 95% confidence intervals through a multivariate analysis of binary logistic regression, where the dependent variable was depression (score at BDI ≥14 points) or anxiety (score at HAS ≥6 points) and the independent variables were gender, marital status, working position, smoking, alcohol use, BDI, and HAS score. Statistical analysis was performed using IBM SPSS Statistics for Windows, Version 29 (Released 2022; IBM Corp., Armonk, New York, United States).

## Results

Among the 232 workers surveyed who relatively met all the selection criteria from the UMF, no worker left the study and there were no incomplete questionnaires. Of all the subjects included, 54.7% (127 workers) were women, and the mean age of the workers was 41 ± 8.6 years. Regarding working conditions, the average seniority at IMSS (in any unit) was 16 ± 2.4 years. However, within the unit, the average time was 9 ± 1.8 years. Despite the relatively short time in the UMF, 91.4% (212 workers) are permanent staff. The nursing staff category had the greatest representation, with 39.7% (92 workers), followed by medical personnel with 34.9% (81 workers). Concerning the sociodemographic and anthropometric variables, the average number of children per worker was 1.69 ± 1.3. Seventy-eight percent (181 workers) of the staff had a bachelor's degree or above, 62.5% (145 workers) had a stable partner (with and without civil partnership), the average body mass index (BMI) of the workers was 27.7 ± 4.2 kg/m^2^, 29.2% of the workers were sedentary, and 51.7% (120 workers) practiced mild physical activity, and the average score for the socioeconomic level tool was of 8.9 ± 1.9 which corresponds to the upper-middle stratum (second highest quintile). Regarding cigarette and alcohol use, the prevalences were 18.5% (43 workers) and 21.6% (50 workers), respectively. The tools used to explore mental disorders showed that the mean scores for all workers surveyed were 12.93 ± 10.2 for HAS. The stratified tool showed 42.2% (98 workers) of the workers with minor anxiety and 33.6% (78 workers) with major anxiety. BDI was 8.08 ± 7.4. Similarly, when stratified, 19% (44 workers) have some level of depression.

Table 1 shows the sociodemographic characteristics according to occupation and job category; there was no relevant difference in gender or marital status. Medical personnel are those who have the highest level of education.

**Table 1:** Demographic and occupational characteristics of healthcare staff *Unmarried category included widowed and divorced participants

Characteristic	Occupation			Working position	
	Clinical Staff		Non-Clinical Staff	Permanent Staff	Non-permanent Staff
	Physician (Family doctor)	Nurse			
	n (%)	n (%)	n (%)	n (%)	n (%)
All	81 (100)	93 (100)	59 (100)	213 (100)	20 (100)
Gender					
Male	39 (48)	39 (42)	28 (47)	94 (44)	12 (60)
Female	42 (52)	54 (58)	31 (53)	119 (56)	8 (40)
Marital status					
Married	43 (53)	69 (74)	34 (58)	138 (65)	8 (40)
Unmarried*	38 (47)	24 (26)	25 (42)	75 (35)	12 (60)
Education level					
Elementary school or less	0 (0)	1 (1)	1 (2)	1 (1)	1 (5)
Middle School	1 (1)	0 (0)	1 (2)	1 (1)	1 (5)
High School	0 (0)	31 (33)	16 (27)	39 (18)	8 (40)
Bachelor’s degree	16 (20)	57 (62)	36 (61)	102 (48)	7 (35)
Master’s degree or more	64 (79)	4 (4)	5 (8)	70 (32)	3 (15)
Previous illness or special health condition					
None	48 (59)	61 (66)	39 (66)	133 (62)	15 (75)
Pregnancy and puerperium	0 (0)	3 (3)	1 (2)	3 (1)	1 (5)
Diabetes Mellitus	1 (1)	8 (9)	1 (2)	10 (5)	0 (0)
Diabetes and other	2 (2)	3 (3)	2 (3)	6 (3)	1 (5)
Arterial hypertension	8 (10)	8 (9)	7 (12)	22 (10)	1 (5)
Arterial hypertension and other	3 (4)	1 (1)	0 (0)	4 (2)	0 (0)
Other	19 (23)	9 (10)	9 (15)	35 (16)	2 (10)
Smoking					
No	74 (91)	75 (81)	41 (69)	174 (82)	16 (80)
Yes	7 (9)	18 (19)	18 (31)	39 (18)	4 (20)
Frequent alcohol consumption					
No	66 (81)	75 (81)	42 (71)	170 (80)	13 (65)
Yes	15 (19)	18 (19)	17 (29)	43 (20)	7 (35)
Frequency of physical activity					
Sedentary lifestyle	25 (31)	31 (33)	12 (20)	63 (30)	5 (25)
Mild activity	39 (48)	48 (52)	34 (58)	112 (53)	9 (45)
Moderate activity	13 (16)	14 (15)	10 (17)	31 (15)	6 (30)
Intense activity	4 (5)	0 (0)	3 (5)	7 (3)	0 (0)
Socioeconomic level					
High stratum	14 (17)	0 (0)	2 (3)	16 (8)	0 (0)
Upper middle stratum	56 (69)	39 (42)	34 (58)	123 (58)	6 (30)
Lower middle stratum	7 (9)	48 (52)	21 (36)	66 (31)	10 (50)
Working class	4 (5)	6 (6)	2 (3)	8 (4)	4 (20)
Hamilton Anxiety Scale					
No anxiety (0-5 points)	22 (27)	27 (29)	8 (14)	50 (23)	7 (35)
Minor anxiety (6-14 points)	34 (42)	37 (40)	27 (46)	95 (45)	3 (15)
Major anxiety (15 or more points)	25 (31)	29 (31)	24 (41)	68 (32)	10 (50)
Beck Depression Inventory					
No depression (0-13 points)	68 (84)	81 (87)	40 (68)	175 (82)	14 (70)
Mild depression (14-19 points)	8 (10)	9 (10)	7 (12)	21 (10)	3 (15)
Moderate depression (20-28 points)	3 (4)	3 (3)	11 (19)	14 (7)	3 (15)
Severe depression (29 or more points)	2 (2)	0 (0)	1 (2)	3 (1)	0 (0)

The sociodemographic and work characteristics, as well as the personal background of the workers, showed significant differences in the screening tests of the tools categorized by diagnosis, non-permanent personnel within the work position, and tobacco use and higher socioeconomic levels were relevant in those with some degree of anxiety. For those who experienced some degree of depression, the association was linked with occupation, less physical activity, and high socioeconomic level. All the associations of the variables are described in Table 2.

**Table 2:** Levels of depression and anxiety according to gender and demographic variables, comorbidity, alcohol-tobacco use, and physical activity *p-value less than 0.05. All hypothesis tests were two-tailed. **Unmarried category included widowed and divorced participants The comparison of proportions was carried out using the chi-square test.

Characteristic	Hamilton Anxiety Scale			Beck Depression Inventory			
	No anxiety (0-5 points)	Minor anxiety (6-14 points)	Major anxiety (15 or more points)	No depression (0-13 points)	Mild depression (14-19 points)	Moderate depression (20-28 points)	Severe depression (29 or more points)
	n (%)	n (%)	n (%)	n (%)	n (%)	n (%)	n (%)
All	57 (100)	98 (100)	78 (100)	189 (100)	24 (100)	17 (100)	3 (100)
Gender							
Male	29 (50.9)	42 (42.7)	35 (44.9)	87 (46.03)	12 (50)	6 (35.29)	1 (33.3)
Female	28 (49.1)	56 (57.3)	43 (55.1)	102 (53.97)	12 (50)	11 (64.71)	2 (66.7)
p-value	0.62			0.77			
Education level							
Elementary school or less	0 (0)	1 (1.1)	1 (1.3)	1 (0.5)	0 (0)	1 (5.9)	0 (0)
Middle School	0 (0)	0 (0)	2 (2.6)	1 (0.5)	0 (0)	1 (5.9)	0 (0)
High School	13 (22.8)	13 (13.3)	21 (26.9)	36 (19)	8 (33.3)	2 (11.7)	1 (33.3)
Bachelor’s degree	20 (35.1)	53 (54)	36 (46.1)	87 (46)	12 (50)	10 (58.8)	0 (0)
Master’s degree or more	24 (42.1)	31 (31.6)	18 (23.1)	64 (34)	4 (16.7)	3 (17.7)	2 (66.7)
p-value	0.05			0.06			
Marital status							
Married	34 (59.6)	61 (62.2)	51 (65.4)	121 (64)	14 (58.3)	9 (52.9)	2 (66.7)
Unmarried**	23 (40.4)	37 (37.8)	27 (34.6)	68 (36)	10 (41.7)	8 (47.1)	1 (33.3)
p-value	0.78			0.79			
Working position							
Permanent Staff	50 (87.7)	95 (96.9)	68 (87.2)	175 (92.6)	21 (87.5)	14 (18.4)	3 (100)
Non-permanent Staff	7 (12.3)	3 (3.1)	10 (12.8)	14 (7.4)	3 (12.5)	3 (17.6)	0 (0)
p-value	0.04*			0.41			
Occupation							
Physician (Family doctor)	22 (38.6)	34 (34.7)	25 (32.1)	68 (35.9)	8 (33.3)	3 (17.7)	2 (66.7)
Nurse	27 (47.4)	37 (37.8)	29 (37.1)	81 (42.9)	9 (37.5)	3 (17.7)	0 (0)
Non-Clinical Staff	8 (14)	27 (27.5)	24 (30.8)	40 (21.2)	7 (29.2)	11 (64.6)	1 (33.3)
p-value	0.24			0.01*			
Previous illness or special health condition							
None	40 (70.2)	67 (68.4)	41 (52.6)	121 (64)	14 (58.3)	11 (64.7)	2 (66.7)
Pregnancy and puerperium	3 (5.3)	0 (0)	1 (1.3)	3 (1.6)	0 (0)	1 (5.9)	0 (0)
Diabetes Mellitus	2 (3.5)	2 (2.1)	6 (7.7)	8 (4.2)	1 (4.2)	1 (5.9)	0 (0)
Diabetes and other	0 (0)	4 (4.1)	3 (3.9)	5 (2.6)	1 (4.2)	1 (5.9)	0 (0)
Arterial hypertension	3 (5.2)	10 (10.2)	10 (12.8)	19 (10)	3 (12.5)	0 (0)	1 (33.3)
Arterial hypertension and other	0 (0)	1 (1)	3 (3.9)	3 (1.6)	1 (4.2)	0 (0)	0 (0)
Other	9 (15.8)	14 (14.2)	14 (17.8)	30 (16)	4 (16.6)	3 (17.6)	0 (0)
p-value	0.08			0.96			
Frequent alcohol consumption							
No	48 (84.2)	80 (81.6)	55 (70.5)	149 (78.8)	16 (66.7)	15 (88.2)	3 (100)
Yes	9 (15.8)	18 (18.4)	23 (29.5)	40 (21.2)	8 (33.3)	2 (11.8)	0 (0)
p-value	0.09			0.28			
Smoking							
No	53 (93)	83 (84.7)	54 (69.2)	156 (82.5)	18 (75)	14 (82.4)	2 (66.7)
Yes	4 (7)	15 (15.3)	24 (30.8)	33 (17.5)	6 (25)	3 (17.6)	1 (33.3)
p-value	0.01*			0.74			
Frequency of physical activity							
Sedentary lifestyle	17 (29.8)	32 (32.7)	19 (24.4)	58 (30.7)	5 (20.8)	5 (29.4)	0 (0)
Mild activity	27 (47.4)	51 (52)	43 (55.1)	99 (52.3)	12 (50)	10 (58.8)	0 (0)
Moderate activity	11 (19.3)	11 (11.2)	15 (19.2)	26 (13.8)	7 (29.2)	1 (5.9)	3 (100)
Intense activity	2 (3.5)	4 (4.1)	1 (1.3)	6 (3.2)	0 (0)	1 (5.9)	0 (0)
p-value	0.55			0.01*			
Socioeconomic level							
High stratum	2 (3.5)	3 (3.1)	11 (14.1)	8 (4.2)	4 (16.7)	3 (17.6)	1 (33.3)
Upper middle stratum	33 (57.9)	66 (67.4)	30 (38.5)	110 (58.2)	11 (45.8)	7 (41.2)	1 (33.3)
Lower middle stratum	17 (29.8)	26 (26.4)	33 (42.3)	64 (33.9)	6 (25)	5 (29.4)	1 (33.4)
working class	5 (8.8)	3 (3.1)	4 (5.1)	7 (3.7)	3 (12.5)	2 (11.8)	0
p-value	0.01*			0.03*			

The categorical variables and their statistical differences regarding the total score of the tools are shown in Table 3. The mean score for the anxiety tool was higher in non-clinical personnel and those who smoke, consume alcohol, and have a high socioeconomic level. Regarding the score of the depression instrument, the highest score was found in those with a high economic level.

**Table 3:** Comparison of age, job tenure, anxiety, and depression scores *p-value less than 0.05. All hypothesis tests were two-tailed. **Unmarried category included widowed and divorced participants All categorical values are robust to normality. Comparison of means between two independent groups performed using Student’s t-test. ANOVA was performed for comparison of three or more independent groups.

Characteristics		Hamilton Anxiety Scale	Beck Depression Inventory	Age	BMI
		Mean ± Standard Deviation	Mean ± Standard Deviation	Mean ± Standard Deviation	Mean ± Standard Deviation
Total Score or Total in sample		12.9 ± 10.2	8 ± 7.5	40.6 ± 8.7	27.8 ± 4.2
Gender	Male	12.4 ± 10.3	7.9 ± 6.9	40.9 ± 8.8	28.4 ± 4.1
	Female	13.4 ± 10.1	8.2 ± 7.9	40.5 ± 8.6	27.3 ± 4.3
	p-value	0.49	0.78	0.72	0.05
Education level	Elementary school or less	14.5 ± 12	10.5 ± 13.4	29.5 ± 0.7	25.2 ± 0.7
	Middle School	26.5 ± 7.8	16 ± 7.1	47 ± 8.5	29.5 ± 0.5
	High School	14.5 ± 11	9.1 ± 6.8	38.4 ± 8.2	28.2 ± 4.3
	Bachelor’s degree	12.8 ± 9.1	7.7 ± 7	39.3 ± 8.1	28.2 ± 4.8
	Master’s degree or more	11.8 ± 11	7.7 ± 8.3	44.3 ± 8.7	26.9 ± 3.3
	p-value	0.22	0.44	0.01*	0.19
Marital status	Married	12.8 ± 9.9	7.9 ± 7.4	41.6 ± 7.7	27.9 ± 4.1
	Unmarried**	13.1 ± 10.6	8.4 ± 7.6	39.06 ± 9.9	27.6 ± 4.4
	p-value	0.83	0.66	0.03*	0.69
Working position	Permanent Staff	12.5 ± 9.6	7.8 ± 7.5	41.4 ± 8.3	28 ± 4.1
	Non-permanent Staff	17.5 ± 14.9	10.8 ± 6.7	32.4 ± 8.5	25.2 ± 5.1
	p-value	0.46	0.28	0.01*	0.86
Occupation	Physician (Family doctor)	12.9 ± 10.9	7.9 ± 8	43.9 ± 8.03	27.7 ± 3.9
	Nurse	11.8 ± 9.8	6.7 ± 5.8	39 ± 7.9	27.6 ± 3.9
	Non-Clinical Staff	14.6 ± 9.8	10.1 ± 8.5	38.8 ± 9.4	28 ± 5.2
	p-value	0.04*	0.08	0.01*	0.01*
Smoking	No	12.2 ± 10.1	7.9 ± 7.5	41.3 ± 8.7	27.5 ± 4.2
	Yes	16.4 ± 9.9	8.7 ± 7.1	37.8 ± 8.2	28.9 ± 4.3
	p-value	0.01*	0.52	0.02*	0.05
Frequent alcohol consumption	No	12 ± 9.6	8 ± 7.7	41.4 ± 8.8	27.8 ± 4.2
	Yes	16.3 ± 11.6	8.2 ± 6.6	37.7 ± 7.5	27.5 ± 4.5
	p-value	0.01*	0.86	0.01*	0.65
Frequency of physical activity	Sedentary lifestyle	11.9 ± 10.1	7.6 ± 6.4	42.4 ± 8.9	27.9 ± 4.5
	Mild activity	13.1 ± 9.9	7.8 ± 0.7	41.1 ± 8.2	27.7 ± 4
	Moderate activity	14.1 ± 10.6	10.2 ± 10.4	36.7 ± 8.9	27.9 ± 4.7
	Intense activity	13.7 ± 14.4+5	6.7 ± 10	37.6 ± 6.1	26.4 ± 3.8
	p-value	0.76	0.3	0.01*	0.8
Socioeconomic level	High stratum	18.8 ± 8.6	14 ± 11.1	42 ± 5.2	29.2 ± 4.5
	Upper middle stratum	11.5 ± 9.5	7.1 ± 6.5	41.4 ± 9.2	27.3 ± 4
	Lower middle stratum	14.5 ± 11.3	7.7 ± 7.2	39.9 ± 8.2	28.3 ± 4.5
	working class	10.5 ± 8.4	13.6 ± 7.5	35.4 ± 7.9	26.9 ± 3.7
	p-value	0.01*	0.01*	0.1	0.18

In the correlation matrix, the total scores for the screening tools were used. Statistically significant differences were found between the HAS score and the BMI (ρ = 0.16) and the BDI score (ρ = 0.57). The BDI score showed a correlation with HAS (ρ = 0.57). The rest of the correlations for all the continuous variables are described in Table 4.

**Table 4:** Correlation matrix for depression and anxiety scores and categorical variables *p-value less than 0.05. The Spearman rho correlation coefficient test was used. All hypothesis tests were two-tailed. UMF: Unidad de Medicina Familiar; IMSS: Instituto Mexicano del Seguro Social

Variable		Age	Work seniority at IMSS (Months)	Work seniority at UMF (Months)	BMI	Hamilton Anxiety Scale (Score)	Beck Depression Inventory (Score)	Socioeconomic level
Age	Correlation coefficient	1	0.71	0.59	0.21	-0.05	0.004	-0.150
	p-value		0.01*	0.01*	0.01*	0.5	0.96	0.02*
Work seniority at the IMSS (Years)	Correlation coefficient	0.71	1	0.83	0.15	-0.05	-0.05	-0.12
	p-value	0.01*		0.01*	0.03*	0.45	0.41	0.08
Work seniority at the UMF (Years)	Correlation coefficient	0.59	0.83	1	0.08	-0.10	-0.12	-0.04
	p-value	0.01*	0.01*		0.20	0.13	0.07	0.51
BMI	Correlation coefficient	0.21	0.15	0.08	1	0.17	0.14	0.03
	p-value	0.01*	0.03*	0.20		0.01*	0.04*	0.69
Hamilton Anxiety Scale (Score)	Correlation coefficient	-0.05	-0.05	-0.10	0.17	1	.57	0.02
	p-value	0.5	0.45	0.13	0.01*		0.01*	0.80
Beck Depression Inventory (Score)	Correlation coefficient	0.01	-0.05	-0.12	0.14	0.57	1	0.07
	p-value	0.96	0.41	0.07	0.04*	0.01*		0.33
Socioeconomic level	Correlation coefficient	-0.15	-0.12	-0.04	0.03	0.02	0.07	1
	p-value	0.02*	0.08	0.51	0.69	0.80	0.33	

Finally, in the survey applied to workers that explores the attitude, knowledge, needs, and fears about COVID-19, being categorized by the degrees of anxiety and depression, we observed that there were workers who considered they were absent as cases increased; they did not consider they had enough knowledge, and they did not believe that the UMF is prepared to serve the population. They also described their fears of the disease, which included workers' fears, the most prevalent being the risk of infecting their family. The answers to these items are described in Table 5.

**Table 5:** Severity categories for depression and anxiety distributed by questions asked to staff and comparison test 1: Totally disagree + partially disagree + neither agree nor disagree, 2: partially agree + totally agree. *P value less than 0.05. All hypothesis tests were two-tailed. The comparison of proportions was carried out using the chi-square test. UMF: Unidad de Medicina Familiar; IMSS: Instituto Mexicano del Seguro Social

Characteristic	Hamilton Anxiety Scale			Beck Depression Inventory			
	No anxiety (0-5 points)	Minor anxiety (6-14 points)	Major anxiety (15 or more points)	No depression (0-13 points)	Mild depression (14-19 points)	Moderate depression (20-28 points)	Severe depression (29 or more points)
	n %	n %	n %	n %	n %	n %	n %
All	57 (100)	98 (100)	78 (100)	189 (100)	24 (100)	17 (100)	3 (100)
I have sufficient information about signs and symptoms of COVID-19							
1	2 (4)	8 (8)	7 (9)	12 (6)	1 (4)	2 (12)	2 (66.7)
2	55 (96)	90 (92)	71 (91)	177 (94)	23 (96)	15 (88)	1 (33.3)
p-value	0.44			0.01*			
I have sufficient information about prognosis of COVID-19							
1	4 (7)	8 (8)	7 (9)	14 (7)	1 (4)	2 (12)	2 (66.7)
2	53 (93)	90 (92)	71 (91)	175 (93)	23 (96)	15 (88)	1 (33.3)
p-value	0.92			0.01*			
I have sufficient information about treatment of COVID-19							
1	3 (5)	16 (16)	10 (13)	20 (11)	3 (12)	4 (24)	2 (66.7)
2	54 (95)	82 (84)	68 (87)	169 (89)	21 (88)	13 (76)	1 (33.3)
p-value	0.13			0.01*			
I have sufficient information about routes of transmission of COVID-19							
1	5 (9)	12 (12)	8 (10)	21 (11)	1 (4)	1 (6)	2 (66.7)
2	52 (91)	86 (88)	70 (90)	168 (89)	23 (96)	16 (94)	1 (33.3)
p-value	0.79			0.01*			
I have sufficient information about precautionary measures of COVID-19							
1	4 (7)	11 (11)	9 (12)	19 (10)	2 (8)	1 (6)	2 (66.7)
2	53 (93)	87 (89)	69 (88)	170 (90)	22 (92)	16 (94)	1 (33.3)
p-value	0.64			0.01*			
The IMSS has provided me with sufficient information about COVID-19							
1	3 (5)	13 (13)	21 (27)	28 (15)	3 (12)	4 (24)	2 (66.7)
2	54 (95)	85 (87)	57 (73)	161 (85)	21 (88)	13 (76)	1 (33.3)
p-value	0.02*			0.08			
The information provided by the IMSS about COVID-19 has been clear							
1	5 (9)	14 (14)	19 (24)	26 (14)	3 (12)	7 (41)	2 (66.7)
2	52 (91)	84 (86)	59 (76)	163 (86)	21 (88)	10 (59)	1 (33.3)
p-value	0.04*			0.01*			
I do not require more information about COVID-19							
1	10 (18)	21 (21)	30 (38)	39 (21)	7 (29)	12 (71)	3 (100)
2	47 (82)	77 (79)	48 (62)	150 (79)	17 (71)	5 (29)	0 (0)
p-value	0.01*			0.01*			
I restrict my social contact because I consider myself dangerous							
1	6 (11)	18 (18)	22 (28)	30 (16)	7 (29)	7 (41)	2 (66.7)
2	51 (89)	80 (82)	56 (72)	159 (84)	17 (71)	10 (59)	1 (33.3)
p-value	0.04*			0.01*			
I feel like my family and friends avoid me because of my job							
1	31 (54)	56 (57)	29 (37)	95 (50)	9 (37)	10 (59)	2 (66.7)
2	26 (46)	42 (43)	49 (63)	94 (50)	15 (63)	7 (41)	1 (33.3)
p-value	0.02*			0.50			
I am afraid of getting sick from COVID-19 and have looked for excuses not to go to work							
1	42 (74)	54 (55)	38 (49)	109 (58)	13 (54)	10 (59)	2 (66.7)
2	15 (26)	44 (45)	40 (51)	80 (42)	11 (46)	7 (41)	1 (33.3)
p-value	0.02*			0.97			
If cases increase, I will miss work							
1	50 (88)	80 (82)	54 (69)	155 (82)	14 (58)	13 (76)	2 (66.7)
2	7 (12)	18 (18)	24 (31)	34 (18)	10 (42)	4 (24)	1 (33.3)
p-value	0.02*			0.06			
I am afraid of COVID-19							
1	31 (54)	35 (36)	28 (36)	77 (41)	11 (46)	4 (24)	2 (66.7)
2	26 (46)	63 (64)	50 (64)	112 (59)	13 (54)	13 (76)	1 (33.3)
p-value	0.04*			0.37			
If I get sick with COVID-19, I will have a serious form of the disease							
1	31 (54)	42 (43)	30 (38)	79 (42)	15 (62)	7 (41)	2 (66.7)
2	26 (46)	56 (57)	48 (62)	110 (58)	9 (38)	10 (59)	1 (33.3)
p-value	0.17			0.22			
I am more susceptible to having COVID-19 sequel							
1	19 (33)	25 (26)	26 (33)	50 (26)	12 (50)	6 (35)	2 (66.7)
2	38 (67)	73 (74)	52 (67)	139 (74)	12 (50)	11 (65)	1 (33.3)
p-value	0.01*			0.57			
COVID-19 is difficult to treat							
1	23 (40)	34 (35)	29 (37)	66 (35)	13 (54)	5 (29)	2 (66.7)
2	34 (60)	64 (65)	49 (63)	123 (65)	11 (46)	12 (71)	1 (33.3)
p-value	0.44			0.04*			
I think the UMF can deal with COVID-19							
1	6 (11)	12 (12)	17 (22)	26 (14)	4 (17)	3 (18)	2 (66.7)
2	51 (89)	86 (88)	61 (78)	163 (86)	20 (83)	14 (82)	1 (33.3)
p-value	0.78			0.18			
We should have more psychological support from the IMSS							
1	52 (91)	87 (89)	63 (81)	165 (87)	19 (79)	15 (88)	3 (100)
2	5 (9)	11 (11)	15 (19)	24 (13)	5 (21)	2 (12)	0 (0)
p-value	0.12			0.08			
What worries me most about COVID-19 is:							
Isolation	2 (4)	1 (1)	2 (3)	5 (3)	0 (0)	0 (0)	0 (0)
Risk of infecting my family	47 (83)	75 (77)	53 (68)	143 (76)	16 (66.7)	14 (82)	2 (66.7)
Risk of becoming seriously ill	3 (5)	11 (11)	8 (10)	17 (9)	3 (12.3)	1 (6)	1 (33.3)
Not counting skills to treat the disease	5 (8)	11 (11)	15 (19)	24 (12)	5 (21)	2 (12)	0 (0)
p-value	0.34			0.8			

Binary logistic regression models were used, where the screening tools were separated into dichotomous results, in which smoking was found to be a risk factor for anxiety (OR 4 95% CI 1.3-12.7). A higher score on HAS (OR 1.07 95% CI 1.04-1.11) and not being permanent staff (OR 3.34 95% CI 1.2-9.3) were found to be risk factors for depression. The models are described in Table 6.

**Table 6:** Risk factors for anxiety and depression *P value less than 0.05 **R^2^ of Cox and Snell 0.188, R^2^ Nagelkerke 0.28 *** R^2^ of Cox and Snell 0.24, R^2^ Nagelkerke 0.38 Binary logistic regression analysis was used. UMF: Unidad de Medicina Familiar; IMSS: Instituto Mexicano del Seguro Social

Characteristic	B	Sig.	OR	95% C.I.	
				Lower	Upper
Dependent variable: Hamilton Anxiety Scale (≥6 points)**					
Gender (Male)	0.66	0.07	1.94	0.96	3.94
Marital status (Married)	-0.18	0.62	0.83	0.41	1.69
Working position (Permanent Staff)	-1.22	0.06	0.29	0.09	1.03
Smoking (Yes)	1.39	0.02*	4.01	1.27	12.73
Frequent alcohol consumption (Yes)	0.64	0.17	1.89	0.76	4.71
Beck Depression Inventory (Score)	0.22	0.01*	1.25	1.14	1.37
Dependent variable: Beck Depression Inventory (≥14 points)***					
Gender (Male)	-0.16	0.73	0.86	0.36	2.05
Marital status (Married)	0.52	0.21	1.68	0.74	3.81
Smoking (Yes)	-0.16	0.76	0.85	0.29	2.43
Frequent alcohol consumption (Yes)	-0.4	0.43	0.67	0.25	1.79
Hamilton Anxiety Scale (Score)	0.07	0.01*	1.07	1.04	1.11
Work seniority at IMSS (Months)	0.02	0.15	1.02	0.99	1.04
Work seniority at UMF (Months)	-0.03	0.22	0.97	0.92	1.02
Working position (Permanent Staff)	0.64	0.25	1.89	0.64	5.58
Working position (Non-Permanent Staff)	1.21	0.02*	3.34	1.19	9.33

## Discussion

Despite the various measures taken by the IMSS, healthcare workers showed a high prevalence of mental disorders (75.6% anxiety, and 18.9% depression), which may explain why interventions were not identifiable or affordable for workers or they became insufficient [19]. Something relevant about this work is that the workers consider that the UMF has provided them with enough information about the disease, however, they are afraid of the disease, of suffering from a serious form, of infecting others, and to a lesser extent of isolation.

The identified prevalence in healthcare workers seemed to be higher than that in the general population, which may be caused by the type of work and that the people had to deal with coronavirus. A study carried out by Li et al., (2020), in China with the support of social networks among the general population, identified an overall prevalence of 11.69% prior to the news of the national dissemination of SARS-CoV-2 (January 20, 2020) and 12.76% after this period [24]. Meanwhile, depression prior to this period was 14.87% and later 15.27% [24]. Our study helps to provide clarity about mental health, showing lower figures before the disease emerged, while when compared with the present study, it is observed that the diseases in the general population are much lower than those among health personnel during the pandemic (75.8% for anxiety and 19% for depression).

Another study, in China, that allows comparison with the general population is the one carried out by Huang and Zhao (2020), which found the prevalence of anxiety as 35.1% and depression as 20.1%, which did not change according to gender, being only higher in those under 35 years old [25]. A much higher prevalence of anxiety can be observed in Mexican workers, even than in those who identified themselves as health workers in China; however, the prevalence of depression in this study was higher than that of the personnel studied (19%), and even greater than that reported by Li et al., (2020), something important to mention is that the studies carried out in China are separated by approximately 30 days, which implies that as the disease and information progress, the mental health of the population begins to suffer consequences [24].

Several papers have reported this aspect among health workers during the COVID-19 pandemic. For example, the study by Lai et al., (2020), carried out in various hospitals in China identified a prevalence of 50.4% depression and 44.6% anxiety, in contrast to our study, which only reported lower depression levels (19%) [26]. Interestingly, Lai et al., (2020) carried out their study during the beginning of the pandemic, so it is assumed that as time went by, new therapeutic, diagnostic, and preventive resources appeared, and in general, more information about the disease. Personnel in Mexico should have a reduction in these alterations, something that apparently turns out to be incorrect. The study also reported differentiated prevalences according to subgroups, with anxiety more frequent in women, which aligned with the findings of the present study (although there was no statistical significance).

Healthcare workers also showed different prevalences according to the type of healthcare unit level where they worked. In this regard, the study by Huang et al., (2020), carried out at a tertiary hospital, found a prevalence of 23.04% for anxiety, with female staff being the most affected (25.67%), as well as a higher score on the anxiety detection instrument in nursing staff [27]. In this study, a lower prevalence than that described by Lai et al. was identified in a similar population, whose difference was the level of care in the health unit where they work [26]. The present study identified a higher prevalence of anxiety in Mexican workers, with women being the most affected. However, the nursing staff was the one that obtained the lowest score in the anxiety instrument, being surpassed by the medical staff and non-clinical staff.

Closer to our population is what was identified by Dosil Santamaría et al., (2020), in their study carried out in Spain in healthcare personnel, in which prevalences of 37% of anxiety and 27.4% of depression were described, and those over 36 years old are the most affected (similar to what was identified by Huang and Zhao (2020), and 44.4% felt fear of contagion and these, in turn, were the population with a higher prevalence rate of mental disorders [25,28]. When compared with what was found in the present study, the prevalence of anxiety continues to be higher in the Mexican population, while depression is much higher in Spanish workers. Interestingly, of the population in this work, 59.65% said they were afraid of COVID-19, and the diagnosis of anxiety was higher (p < 0.01).

In Latin America, we can address these alterations. The study by Magaña-Salazar et al., *(2020)*, carried out in El Salvador on workers at a second-level hospital (which included clinical and non-clinical staff), identified a prevalence of depression of 9.1%, 51.2% had minor anxiety and 13.2% had major anxiety [29]. When compared with the population of the present study, a higher prevalence of major anxiety was observed (33.5%), although it was slightly lower in the anxiety rating (42.1%) and twice the proportion of workers with depression (18.9%). The personnel with the greatest impact on their mental health, according to Magaña-Salazar et al., (2020), were the nursing and non-clinical personnel, something that could not be identified in the Mexican nursing personnel but was the case for the non-clinical personnel [29].

To compare what was found in similar health units and populations, we have the study by Rubio-Morales et al., (2021), carried out in a UMF in Ciudad Obregón, Sonora, Mexico, in which greater anxiety was found in 7.7% of workers, and 6.9% moderate and high depression, something quite different from what was found in the present study, where the prevalence was much higher in a population subjected to the same working conditions [30].

The research we conducted may have some limitations; these biases could stem from workers not being completely honest when answering the surveys (information bias), to mitigate this issue, we ensured anonymity during the survey and invited all workers to participate, thereby avoiding self-selection bias. Furthermore, since the study is cross-sectional, it is difficult to establish causality. However, our study serves as a foundation for future research that considers variables that showed statistical significance in our work. Going forward, the main focus should be on developing mental health interventions and evaluating their effectiveness.

## Conclusions

Diseases that pose a risk in all areas must be addressed by the public health system. It is now the obligation of health institutions to carry out periodic mental health evaluations to identify healthcare workers at risk so that they receive timely and appropriate treatment and prevent inadequate care in the population since the staff is the most important resource of this type of institution.

This work was carried out to emphasize the persistent problem at a UMF; therefore, it is proposed that the management of the unit evaluate the best way to intervene with the workers to improve their mental health because although it is part of the definition of health established by the World Health Organization, the IMSS, which is one of the main institutions in charge of the health of a large proportion of the workers of Mexico, omits the lack of support for the mental health of its workers.
